# Autism and schizophrenia spectrum disorder: phenomenological qualitative study of patients’ experience

**DOI:** 10.3389/fpsyt.2025.1669930

**Published:** 2025-10-08

**Authors:** Aleksandra Jeličić, Maja Drobnič Radobuljac, Louis Sass, Borut Škodlar

**Affiliations:** ^1^ Student Health Centre of the University of Ljubljana, Ljubljana, Slovenia; ^2^ Faculty of Medicine, University of Ljubljana, Ljubljana, Slovenia; ^3^ University Psychiatric Clinic Ljubljana, Ljubljana, Slovenia; ^4^ Rutgers University, The State University of New Jersey, Piscataway, NJ, United States

**Keywords:** autism spectrum disorder, schizophrenia spectrum disorder, first-person perspective, ipseity disorder, intersubjectivity

## Abstract

**Objective:**

Autism spectrum disorder (ASD) and schizophrenia spectrum disorder (SSD) overlap in behavioral signs, particularly in social functioning; consequently, SSD patients are frequently misdiagnosed with ASD and vice versa. The neurodevelopmental and spectrum nature of both disorders, including milder variants, further complicates differential diagnosis, which calls for a better differentiation by looking at the subjective experience of patients. To our knowledge, no prior clinical studies have directly and comparatively examined the subjective experiences of individuals from these two spectra. The present study adopts a phenomenological approach traditionally applied to SSD; it reveals qualitative similarities and differences in these two spectra: in the experience of oneself, the world, and interpersonal relationships.

**Methods:**

The study included 42 participants, aged 15 to 26, all with at least average intelligence and no acute psychiatric symptoms, as verified by the Symptom Checklist (SCL-90-R). We interviewed participants in depth on their experiences and applied the Examination of Anomalous Self-Experience (EASE), and selected parts of the Examination of Anomalous World-Experience (EAWE).

**Results:**

Differences were observed across all five EASE domains, with higher levels in the SSD as compared to ASD in minimal self-disorder, demarcation phenomena, paranoid anxiety, short term memory disorder, and magical thinking. Meanwhile, obsessive thinking, attention problems, diminished presence in the world, social anxiety, and hyper-reflectivity overlapped in both groups. The most significant qualitative overlapping within EAWE were in abnormalities within social interactions, increased auditory perception intensity and synesthesia. Within overlaps important qualitative differences are noted and described.

**Conclusions:**

Despite considerable overlap in outer manifestations, we found important qualitative differences that point to the centrality of a disorder of ipseity in the SSD versus of primary intersubjectivity in ASD.

## 1 Introduction

The concept of autism was introduced by Bleuler in 1911 and defined as a detachment from the outer world and reality (1), accompanied by a predominance of inner fantasy life (2). It was considered a fundamental symptom of schizophrenia; and Bleuler believed that autism existed on a continuum, from less severe cases to extreme withdrawal from the social world (1).

Kanner and Asperger used the term “autistic” to describe children with specific lack of emotional and social engagement (3, 4). Contemporary conceptualization defines autism as a spectrum (ASD), encompassing various disorders with symptoms on a continuum. Similarly, schizophrenia (SSD) is a heterogeneous disorder with variations in symptoms and manifestations. Autistic symptoms are considered an integral part of SSD and significantly affect the poorer functioning of individuals living with schizophrenia (5, 6), although functional difficulties are characteristic of both disorders. Initially, SSD and ASD were conflated into one category, with Kanner considering autism as an early manifestation of schizophrenia (3). Disorders were later delineated into distinct diagnostic entities mostly by Rutter and Kolvin, primarily based on the age of onset (7, 8). Over the past three decades, the neurodevelopmental model of schizophrenia has gained prominence (9). Studies highlight that social deficits in SSD are not merely by-products of the illness but are early-emerging, defining, and persistent characteristics of the disorder, with neural abnormalities preceding formal illness onset (10). Subsequent research has challenged the view that these two entities are entirely unrelated, suggesting they may share underlying pathogenic mechanisms (11, 12) and genotypical features, leading to phenotypical and endophenotypic overlaps (13, 14). A meta-analytic work including 19 different studies comparing social-cognitive performances in subjects with SSD and subjects with ASD, showed how social cognition deficits are similar in the two disorders: no significant differences in the Theory of Mind, emotional intelligence, and social skills tests were observed (15). Research indicates that the overlap is most pronounced in negative symptoms (16), disorganization, attention to detail, and imagination, with deficient social skills more prominent in ASD and positive symptoms more prominent in SSD (17). Similar patterns of overlaps and differences between SSD and ASD have been observed and reported in children (18–20). Several studies showed that subjects with a childhood diagnosis of autism are frequently diagnosed with a SSD during adolescence and early adulthood (21–25). Some studies also observed that the presentation of early onset schizophrenia in younger patients, especially before the onset of hallucinations and/or delusions, is difficult to clinically differentiate from ASD (26). It has been found that ASD occurs, on average, in 24% of people with SSD (27), and in 5% of people with first episode psychosis (FEP) (28).

Most studies considering the two disorders have focused on behavioral signs, leading to varied interpretations of the spectra as either identical, distinct, or somewhere in-between (29). If we focus solely on behavioral signs and classical symptoms of the disorders, we have no insight about the quality of experiences and perceptions of people with SSD or ASD. Phenomenological research and literature on SSD have emphasized minimal self-disturbance or ipseity disturbance (involving pre-reflective and implicit levels of selfhood) as key features in SSD (30, 31). Alterations of self-experience in schizophrenia also include a failure of transparency of consciousness, which becomes object of intense self-scrutiny, compatible with the key notion of “hyper-reflexivity” and reflective of an exaggerated self-presence (32).

Research shows that ipseity disturbance or minimal self-disorder can reliably differentiate schizotypal disorder from ASD (29). Other studies confirm that the basic self or sense of agency in ASD is intact, which implies that the first-person perspective remains stable (33).

Systematic clinical phenomenological studies of subjective experiences in ASD, focusing on the first-person perspective, are very limited. Most studies exploring the autistic mind focus on deficits in social cognition within neuroscience frameworks and explore symptoms and signs from a third-person perspective (34). While these theories are scientifically validated, none identify a primary psychopathological organizer explaining the logical interaction between ASD symptoms. By focusing solely on behavioral signs without inner psychopathological organizers, the definition of ASD appears overly inclusive and generic (35). Lack of research on the first-person experiential dimension in ASD makes it difficult to identify the key problem in ASD (34).

Phenomenologically oriented authors explain ASD through the theoretical framework of intersubjectivity (36–39) emphasizing primary intersubjectivity deficits as the essential problem in ASD. While both disorders can exhibit difficulty with social interactions, autistic individuals seem to have greater difficulties in the implicit understanding of social situations (39). What is lacking in ASD seems to be a fundamental embodied grounding of social perception and interaction very early in life—that is, in the basic intercorporeality (40) that enable one to directly grasp the subjectivity of the other person. Such persons attempt to compensate this blind spot, which relates to this basic disturbance of bodily being-with-others, by employing intellectual constructs and assumptions about others (36). Systematic study of the subjective experiences of people who live with a particular disorder throughout their lives allows for better insight and understanding of that disorder.

Direct comparison of lived experiences between ASD and SSD can highlight shared and divergent mechanisms, underlying pathways to social dysfunction. Qualitative comparison simultaneously enables specification of what is a core characteristic of a particular disorder, by which the disorders can be more reliably distinguished from one another. The goal of our study is to illuminate these disorder-specific mechanisms. Searching for qualitative differences can be especially important in disorders such as SSD and ASD, which often overlap in signs and symptoms. This is addressed by questionnaires designed to identify each disorder, but can lead to false positive results and consequently incorrect diagnoses.

The primary research hypothesis is that despite the overlap in behavioral signs and symptoms, the disorders differ in the experience of self and the world. The experiential content by which the disorders can be distinguished can be easily addressed during diagnostic interviews, which may represent an important clinical contribution.

## 2 Methods

### 2.1 Participants and procedures

The study included a clinical population of adolescents and young adults, aged 15 to 26. The age of participants covered the period of adolescence and young adulthood — the prodromal phase of psychosis — when young individuals seek psychiatric/psychological help due to emerging difficulties. This is also the time when differential diagnostic dilemmas most often arise: whether it is a prodrome within the SSD spectrum or an unrecognized and decompensated ASD.

Before entering the study, patients had already been diagnosed with either ASD or SSD and were recruited from outpatient clinical settings where they were already receiving treatment.

Patients were recruited from outpatient settings. Participants were not acutely psychotic and had no comorbid mood, organic, or substance-use disorders. They were of at least average intelligence and had no speech disorders. Intellectual abilities were indirectly assessed based on the type of education and general academic performance. To confirm the diagnosis, an additional assessment was performed (upon entry into the study) by clinicians experienced in adolescent and adult psychopathology. If needed, additional psycho-diagnostic assessment was conducted by a clinical psychologist using the Autism Diagnostic Observation Schedule (ADOS-2) (41). Acute psychotic symptomatology was excluded during the interview and with the use of the Symptom Checklist-90-Revised (SCL-90-R) (42). During the study, diagnoses were made according to ICD-10 diagnostic criteria (43). If at least two clinicians reached a consensus that the individual did not have ASD or SSD, the individual was not included in the study. At initial meeting, a psychological exploration of social history and functioning was obtained. The total number of participants included in the study was 42, with 21 individuals in each diagnostic category.

The study sample size was based on a phenomenological or qualitative research recommendations in order to achieve data saturation in focused groups (44). All participants signed a research consent form. The protocol of the study was approved by the Commission of the Republic of Slovenia for Medical Ethics (No.: 0120-361/2018/4).

The study involved one to two individual meetings with patients, with interviews lasting 2 to 4 hours on average. The EASE and partial EAWE interviews were recorded and transcribed. All interviews, except two, were conducted by the first author (AJ), a clinical psychologist experienced in working with both spectra and trained in conducting interviews. Two interviews were conducted by B.Š., who also supervised the interviews and is a co-author of the EAWE.

### 2.2 Measures

To exclude comorbidities involving other acute psychiatric disorders, a *Symptom Checklist (SCL-90-R)* (42) was applied. SCL-90-R is a self-report symptom inventory that assesses current psychological symptom status and can be used with adolescents and adults.

To capture subjective experience, we applied the EASE scale *(Examination of Anomalous Self Experience*) (45) in both samples. The EASE consists of 57 items thematically divided into 5 domains: (1) Cognition and stream of consciousness, (2) Self-awareness and presence, (3) Bodily experiences, (4) Demarcation/Transitivism, and (5) Existential reorientation. All items were rated as “present” (1) or “not present” (0). Additionally, we applied two domains of the EAWE scale (*Examination of Anomalous World Experience*) (46): namely (1) Space and Objects and (3) Other Persons. These two domains were chosen due to the expected prominence of overlapping in these two areas, with the aim of exploring, in more detail, potential subjective differences (within overlapping domains) between the two spectra of disorders. The two EAWE domains correspond to the two domains of symptoms classically described in ASD, namely, altered social perception but also anomalies of perception, especially involving lessening of the unified or Gestalt-like qualities of visual perception (“central coherence disorder”) (47) and hyper- or hypo-reactivity to sensory input.

A difference between the two interview formats is that the EAWE, unlike the EASE, includes items thought to be common in schizophrenia spectrum but not necessarily to differentiate schizophrenia spectrum from certain other conditions (46, 48). Some items and subtypes are included in the EAWE even though they do not seem more characteristic of schizophrenia-spectrum than of certain other abnormal conditions, especially severe affective disorders and forms of paranoia (46–48). It is noteworthy that paranoia can also appear in ASD due to wrong assumptions that the social interactions are intentionally hostile; this can lead to long-term feelings of persecution (49).

Semi-structured interviews allowed for additional exploration of individuals’ experiences, including some additional questions about oneself, their interests, thinking, fantasy and social world. Participants were encouraged to provide examples when describing a particular experience.

### 2.3 Data processing and statistical analyses

Descriptive statistics (frequencies, percentages, averages) were used to describe the sample (diagnoses, age, sex, comorbid disorders, education). The raw results on SCL-90-R were converted to standard T scores and Student’s *T*-test was used to compare individual symptom scales and global indices between the 2 samples.

For interviews (EASE and EAWE), the Mann-Whitney *U* test was used to show differences between the samples on individual EASE domains, while the Students *T-test* was used to compare means of the total EASE score and 2 EAWE domains. Statistical analyses were done in IBM SPSS statistics 22.0.

**Figure 1:**
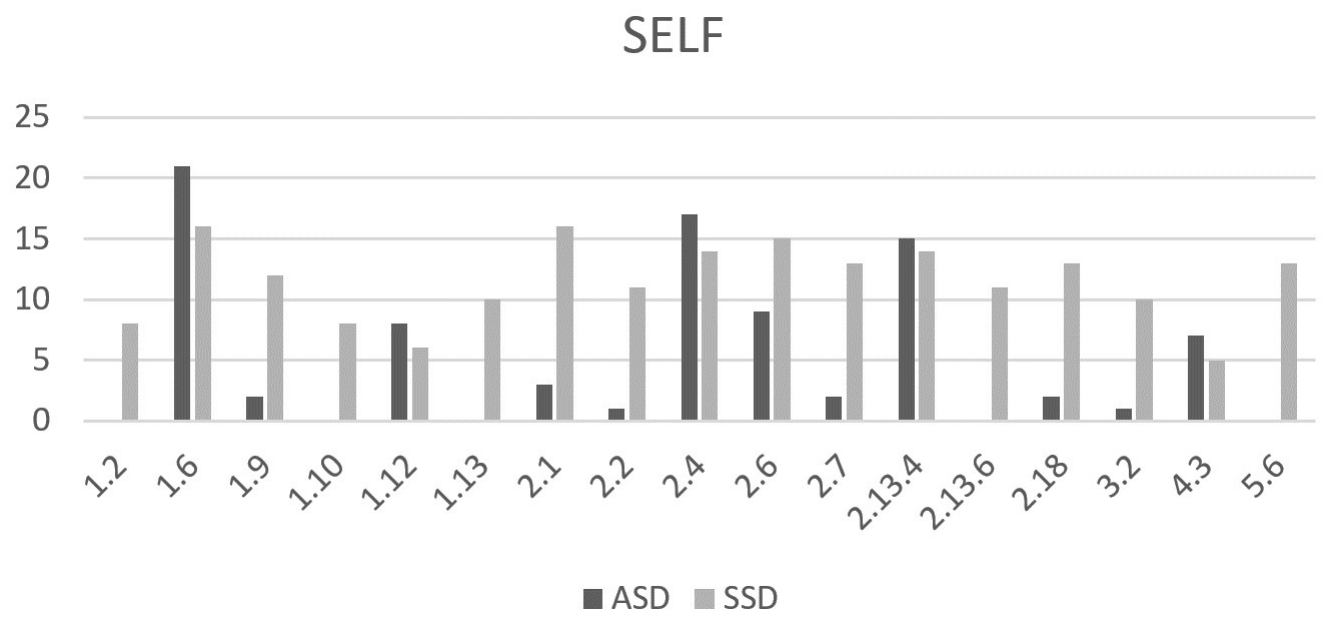
Comparison between the number of individuals with ASD and SSD identified with selected EASE items. 1.2 Loss of thought-ipseity, 1.6 Ruminations-obsessions, 1.9 Ambivalence, 1.10 Inability to discriminate modalities, 1.12 Attentional disturbances, 1.13 Disorder of short-term memory, 2.1 Diminished sense of basic self, 2.2 Distorted first person perspective, 2.4 Diminished presence, 2.6 Hyperreflectivity, 2.7 I-split, 2.13.4 Social anxiety, 2.13.6 Paranoid anxiety, 2.18 Diminished vitality, 3.2 Mirror-related phenomena , 4.3 Threatening bodily contact, 5.6 Magical ideas.

**Figure 2:**
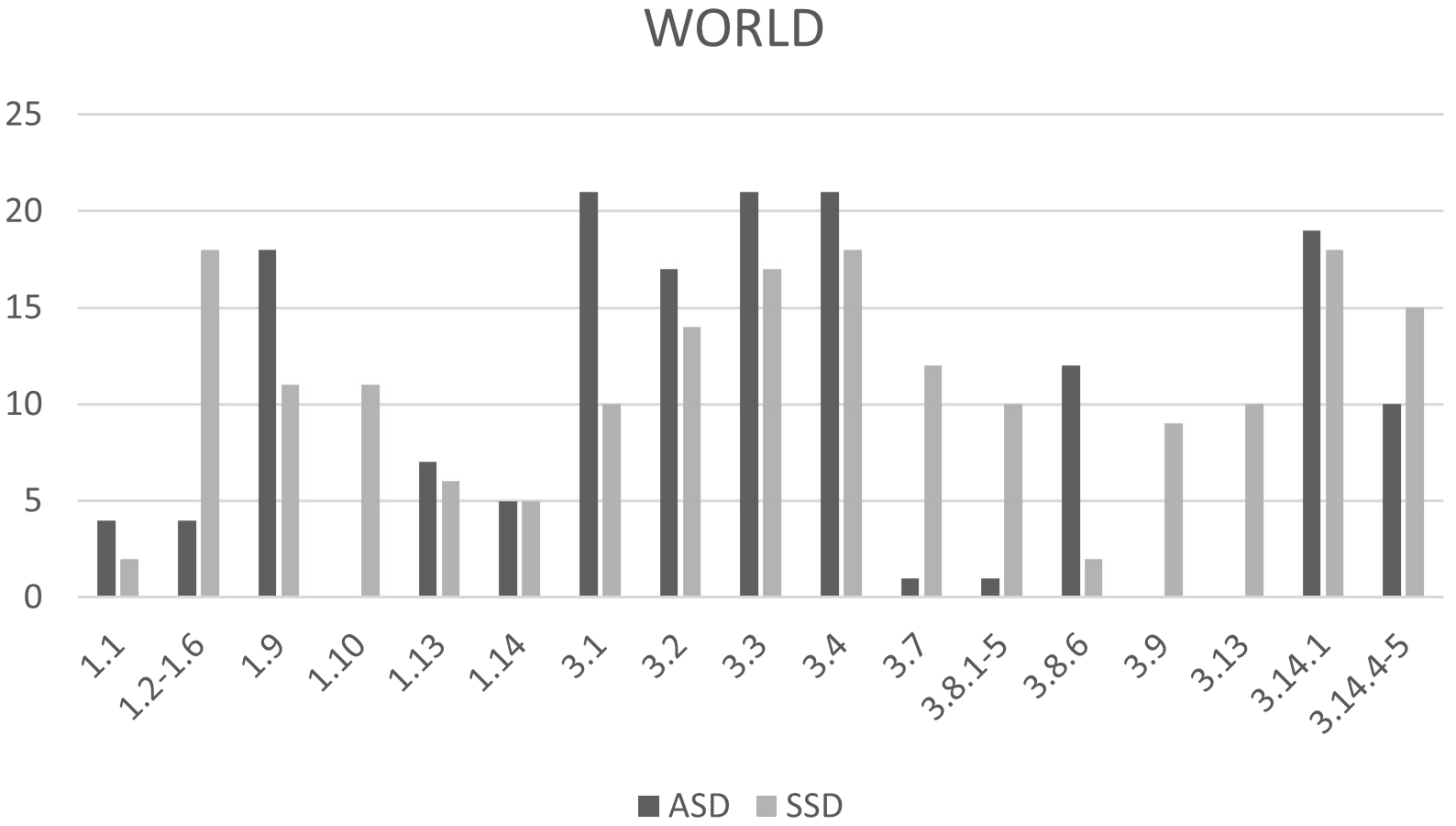
Comparison between the number of individuals with ASD and SSD identified with selected EAWE items. 1.1 Abnormal intensity of visual perception, 1.2-1.6 Visual instability, 1.9 Abnormal intensity of auditory perception, 1.10 Auditory instability, 1.12 Disturbances if other senses, 1.13 Synesthesia, 3.1 Lack of social understanding or interpersonal attunement (hypoattunement), 3.2 Sense of remoteness from others, 3.3 Alienated/intellectual strategies for understanding others, 3.4 Sense of inferiority, criticism, or mistrust in relation to others, 3.7 Disturbance of self-other demarcation, 3.8.1–5 Difficulties with the gaze-”transparency issues”, 3.8.6 Unspecific uneasiness with the gaze, 3.9 Depersonalization of others, 3.13 People seem as if communicating something special or unusual, 3.14.1 Active withdrawal, 3.14.4–5 Compulsive interpersonal harmony and extreme compliance.

The occurrence of individual items within EASE (**Figure 1**) and EAWE (**Figure 2**) domains for both samples is shown in a number of participants who reported the phenomenon or item. Phenomena are categorized according to the domains set within the interviews and according to the categories of study, namely, experiencing oneself, world, and other people. Only the most significant overlaps and deviations within individual items or sub-items, are presented.

The EAWE domain Space and Objects contains 17 items describing various perceptual abnormalities within two main modalities: Visual perceptions (1.1–1.7) and Auditory perceptions (1.9–1.12). Based on the obtained results, and for better clarity, we combined individual phenomena within the sensory modality and divided them in two subcategories: instability of perception (for visual perceptions: 1.2–1.7 and for auditory perceptions: 1.10–1.12) and intensity of perception (for visual perceptions: 1.1 and for auditory perception: 1.9). Content differences between the samples are illustrated with quotations from participants.

## 3 Results

### 3.1 Quantitative results

#### 3.1.1 Sample characteristics

The average age of participants was 19.8 years, with no significant age difference between the samples. There were no significant statistical differences in gender distribution or years of education. All participants attended standard schools, with an average of 12.4 years of schooling, corresponding to the final year of high school in Slovenia. It was not surprising that age at diagnosis was statistically significant, given that individuals with ASD are typically diagnosed earlier than those with SSD. The ASD sample had more comorbid disorders, with the highest number of patients being treated for depression (19%). In the SSD sample, 3 individuals had had an ASD diagnosis in childhood, and 1 had been diagnosed with ADHD.

#### 3.1.2 Symptom check list

Neither sample exhibited elevated acute symptomatology that was clinically significant—consistent with the study’s entry criteria (T values were below average). The difference on the Paranoid Ideation (PAR) scale was statistically significant [for ASD T = 41, for SSD T = 47, *P<*05], although T values were not above average, indicating character traits rather than acute paranoid psychopathology in SSD.

#### 3.1.3 EASE and EAWE scale

On the EASE scale, statistically significant differences were evident across all five domains and in the overall result, with the SSD sample scoring significantly higher than the ASD sample (**Table 1**). On the EAWE scale, by contrast, there was no statistically significant difference in the overall score obtained between the two spectra within either of the selected two domains (**Table 1**). This lack of contrast was not surprising, given that the two selected EAWE domains were chosen precisely because they contain items deemed especially likely to be prominent in ASD as well as in SSD.

**Table 1:** EASE and EAWE domain scores and total EASE score for both samples.

		EASE						EAWE	
		Cognition and stream of consciousness	Self-awareness and presence	Bodily experiences	Demarcation/ Transitivism	Existential re- orientation	Total	Space and Objects	Other Persons
AS	MEAN	2.19	3	0.14	0.38	0.28	5.86	1.86	7.66
	SD	1.03	1.89	0.48	0.59	0.56	2.99	0.96	2.13
	MEDIAN	2	3	0	0	0	5	2	8
	RANGE	0–5	0–2	0–2	0–2	0–4	3–15	0–4	3–12
SS	MEAN	5.52	7.66	1.62	1	1.81	17.24	2.66	8.42
	SD	3.62	4.19	1.36	0.95	1.47	9.38	2.26	4.88
	MEDIAN	5	9	1	1	2	19	2	8
	RANGE	0–13	0–14	0–4	0–4	0–2	0–34	0–8	0–16
	P-value	< .001	< .001	< .001	< .001	< .001	< .001	0,4226	0,5867
	Significant differences	**	**	**	**	**	**	NS	NS

** significant statistical difference.

NS, non-significant statistical difference.

AS, autism spectrum disorder.

SS, schizophrenia spectrum disorder.

### 3.2 Qualitative results

The qualitative findings begin with an exploration of self-experiences reported by participants from both spectra. These accounts include items from the EASE interview, supplemented by additional inquiries into self-related experiences. This is followed by results concerning participants’ perceptions of the external world and their interactions with others, as captured by two EAWE domains: Space and Objects and Other Persons. Key similarities and differences between the two groups are highlighted, with selected experiential phenomena illustrated through direct participant quotations.

#### 3.2.1 Self

The most prominent overlaps between the two groups were observed within the following items: 1.6 Ruminations-obsessions, 1.12 Attentional disturbances, 2.4 Diminished presence, 2.6 Hyper-reflectivity, 2.13.4 Social anxiety, and 4.3 Threatening bodily contact.

*Ruminations* (1.6) were frequently reported by participants in both groups and typically involved repetitive thoughts or re-experiencing of social situations encountered during the day. Within *Attentional disturbances* (1.12) fixation of perception on a specific detail was common across both groups. For example, a person with ASD said, *“Someone in the room wouldn’t notice that the walls are green, they would focus on the conversation, people. I would wonder why these walls are green. Somehow I am more aware of such details.”* Similarly, a person with SSD said, *“When I talk to someone, I can only focus on their fingers and see nothing else.”*

Lasting feelings of barrier or exclusion from the world, capturing by item *Diminished presence* (2.4) was described by individuals from both spectra. An individual with ASD described this feeling: *“Among others, I feel like I stand out from the norm. For example, if there are apples in a basket, and then there’s one pear, one of these things doesn’t belong here, it’s still fruit but it’s not the same.”* The same quality of experience is found in the SSD sample, for example: *“I don’t feel like part of society; it’s like I live a little on the edge of society.”*

*Hyper-reflectivity* (2.6), intense thinking/reflection with reduced spontaneity was also reported in both groups. A participant with ASD said, *“I’m alone in my head and think, for example, why we say good day; such things where answers don’t exist.”* Similarly, a person with SSD said, *“I think a lot about why we talk like this, why we even use language, just because animals don’t have language, they just meow, chirp. I introspect and question about the world for a very long time.”*

Within the *Anxiety* items (2.13) both samples overlapped in the occurrence of social anxiety which relates to feeling of being stressed and uncomfortable among others. *Threatening bodily contact* (4.3) was reported only in subtype 4.3.1 by participants with ASD. This involves intense discomfort or anxiety when touched unexpectedly or when in close physical proximity to others. Example from a person with ASD: *“I don’t like hugs or touches when I don’t expect them. It’s an absolutely unpleasant feeling, like something itches, and no matter how much you scratch, it won’t stop itching.”* SSD group also reported experiences under subtype 4.3.2, which include sensations of annihilation or disappearance in response to physical contact—phenomena not observed in the ASD group.

The most prominent differences between the two samples on EASE were observed in the following items: Diminished Sense of Basic Self (2.1), Distorted first-person perspective (2.2), Ambivalence (1.9), I-split (2.7), Diminished vitality (2.18) and Mirror-related phenomena (3.2).

Most participants in the SSD group reported a *Diminished Sense of Basic Self* (2.1), with 13 individuals experiencing these feelings from adolescence, and 3 since childhood (before age 12). *Distorted first person perspective* (2.2), characterized by a diminished sense of self or a persistent phenomenological distance between the self and experience, was also predominantly described by individuals with SSD. One participant explained: *“It’s like I’m controlling my body from my head, like I’m at a control panel - at some distance from my* sp*eech, from myself.” Mirror-related phenomena (3.2)*, involving bodily instability or perceptual changes, and *Diminished Vitality (2.18)*—particularly the trait-like subtype 2.18.2—were also frequently reported by SSD participants. For example: *“No matter how much I sleep, I never feel alive inside.”*

This kind of profound self-instability, associated with an altered or diminished basic sense of self, was not observed in the ASD group. Individuals with ASD described a strong sense of being fundamentally different from others, without experiencing a loss of self-existence. One participant with ASD shared: *“Since early on, I have felt different from others, like an alien who accidentally landed on Earth.”* In further explorations of self-experience, individuals with ASD often described themselves using traits valued through achievement or external validation, such as being “very intelligent,” “smart,” “honest,” or “disciplined.” Despite a clear awareness of their differences from peers, this did not appear to disrupt their basic sense of self. For instance: *“I know I am different from others, I have a harder time talking, but it is still me. I am very much aware of myself.”* However, many reported a poor social self-image, describing themselves as “always being at the bottom of the social hierarchy” or even as a “social anomaly.” They also expressed difficulty in understanding how they appear to others. As one participant noted: *“The problem is I cannot see myself from outside.”* This lack of perspective-taking often hindered their intentionality and social engagement. One adolescent with ASD explained: *“I don’t know what to do during breaks, what is expected from me, so I just sit in the corner of a school hallway and think about characters from my books.*”

*Ambivalence (1.9)* and *I-split (2.7*)—phenomena commonly described in schizophrenia literature^1^—were also predominantly observed in the SSD group. Several phenomena appeared exclusively in the SSD group, including: *1.2 Loss of thought ipseity*, *1.10 Inability to discriminate modalities, 1.13 Short-term memory disturbances, 2.13.6 Paranoid anxiety, and 5.6 Magical ideation.*

While both groups reported anxiety in social situations, Paranoid Anxiety (2.13.6) was unique to the SSD group. One participant described: “When I am around others I don’t know well, I feel pressure on my psyche and I feel threatened.”

Magical Ideation (5.6) was also reported exclusively by SSD participants and was associated with beliefs in esotericism, reincarnation, occultism, astrology, colors, and faith. In contrast, individuals with ASD denied such beliefs, instead emphasizing a preference for tangible, rational, and logical thinking. As one young person with ASD stated: “Concrete evidence is important to me, and I think strategically, systematically, and practically.”

#### 3.2.2 World: space and objects and other persons

In the domain of perception (*Space and objects*), the most significant overlaps between the two groups were observed in the *abnormal intensity of external stimuli*, both in visual (1.1) and auditory perception (1.9). Notably, a greater number of participants from both groups reported abnormalities in auditory intensity. Some of the participants from both groups also described disturbances in *other sensory modalities* (1.12) and *synesthesia* (1.13).

The most notable difference in the perception of the external world was found in *auditory instability*, which was reported exclusively by participants in the SSD group. These experiences included auditory illusions and hallucinations. Regarding visual instability, many SSD participants described phenomena such as visual illusions and hallucinations, blurred vision, transient blindness, loss of perceptual stability, changes in color perception, macropsia, and altered spatial perception of objects. Individuals with ASD who reported visual illusions or pseudo-hallucinations typically experienced them under conditions of stress.

In the domain *Other Persons* which encompasses social experience, the greatest overlap between the two groups was observed. The most significant overlaps were found within the following items: *3.1 Lack of social understanding or interpersonal attunement*, *3.2 Sense of remoteness from others, 3.3 Alienated/intellectual strategies for understanding others*, *3.4 Sense of inferiority and criticism*, *3.8 Difficulties with the gaze*, *3.14.1 Active withdrawal* and *3.14.4–5 Need for compulsive interpersonal harmony and extreme compliance*. Below we elaborate on these overlaps, while also noting certain differences between the ASD and SSD groups.

All participants in the ASD group identified with difficulties described in items 3.1, 3.2, and 3.4. Most individuals from both groups identified with subcategory *3.1.1, Loss of social common sense.* This refers to the absence of intuitive, embodied social knowledge. For example, one participant with ASD stated: “*I only later realized that people communicate with bodies and have non-verbal language, which I read about.”* A similar sentiment was expressed by a participant with SSD: *“I don’t know how to behave like others. Everything human doesn’t work for me.”* Participants from both groups reported using compensatory strategies such as observing and imitating others’ behavior or reading psychological/philosophical literature to better understand and engage in social situations. These strategies are captured by item 3.3 *Alienated/intellectual strategies for understanding others.*

Sense of inferiority, criticism, or mistrust in relation to others (3.4) was also strongly present in both groups. While Self-consciousness and self-criticism (3.4.1) were common across both samples, Paranoia and pervasive mistrust (3.4.2/3) were more prevalent in the SSD group. Sense of remoteness from others (3.2), which overlaps with EASE item 2.4, was strongly present in both groups. Similarly, Compulsive interpersonal harmony and extreme compliance (3.14.4–5) were frequently reported, often stemming from feelings of inadequacy and a strong desire for social acceptance.

Although both groups exhibited difficulties with eye contact and tendencies toward social withdrawal, our findings revealed important qualitative differences between them. In *Difficulties with gaze* (3.8), the apparent overlap between groups was found to be superficial, with distinct underlying experiences. Participants with SSD reported gaze-related difficulties primarily due to demarcation deficits—feelings of interpersonal transparency, intrusiveness, or exposure (subcategories 3.8.1 and 3.8.2). These experiences were not reported by individuals with ASD. One SSD participant described: *“I don’t look into eyes because it feels like someone is ‘piercing’ me or could see into me.”* In contrast, individuals with ASD described eye contact as feeling unnatural or overly effortful. For example: “*I always thought there must be some rules that teach you the right proportions of how much to look into someone’s eyes.”* Others even questioned its necessity: *“I don’t know why it’s necessary to look into eyes, I only look if I’m interested in the color of the other’s eyes, and otherwise it seems unnecessary for communication.”* Interestingly, many individuals with ASD reported learning eye contact through parental encouragement and acknowledged its social expectation. However, this learned behavior did not necessarily enhance their understanding of others’ subjectivity. As one participant noted: “*As a child, I didn’t have eye contact, now I do, but it’s learned, and I still can’t grasp people’s facial expressions.”*

*Active withdrawal* (3.14.1) was reported by most participants in both groups. For example, one SSD participant said, *“I somehow withdrew from this society.”* Similarly, an individual with ASD shared: *“I prefer to be in my room, in my castle.”* Descriptions of social withdrawal in both groups also reflected a natural preference for solitude. For instance: *“I always liked being alone, even as a child.”* or a statement from a person with SSD: *“I like peace and solitude, even from childhood.”* It is important to note that the need for solitude does not preclude feelings of loneliness, which were also commonly expressed.

A key qualitative difference again emerged in disturbances of self–other demarcation, which appeared exclusively in the SSD group. One participant described: *“Sometimes I get the feeling that others have such intuition that they can influence me, and I have to physically withdraw.”* In contrast, individuals with ASD often withdrew due to the exhaustion caused by the constant effort required to interpret social cues: *“I need a few days without talking to anyone. I need to retreat from the constant effort to understand others and the lack of control.”* Some also cited hypersensitivity to sensory stimuli as a barrier to social interaction.

The most pronounced differences in the experience of others were observed in the following items: *Disturbances of self-other demarcation* (3.7), *Depersonalizations of others* (3.9) and *People seem as if communicating something* sp*ecial or unusual* (3.13) - all of which were reported exclusively by the SSD group. Within item 3.13, the majority (85%) of SSD participants described experiences of paranoid significance (3.13.1). Some also reported Depersonalization of others (3.9), as illustrated by the following quote: *“I experience other people as robots under human skin.”* No participants from the ASD group reported such experiences.

**Figure 3:**
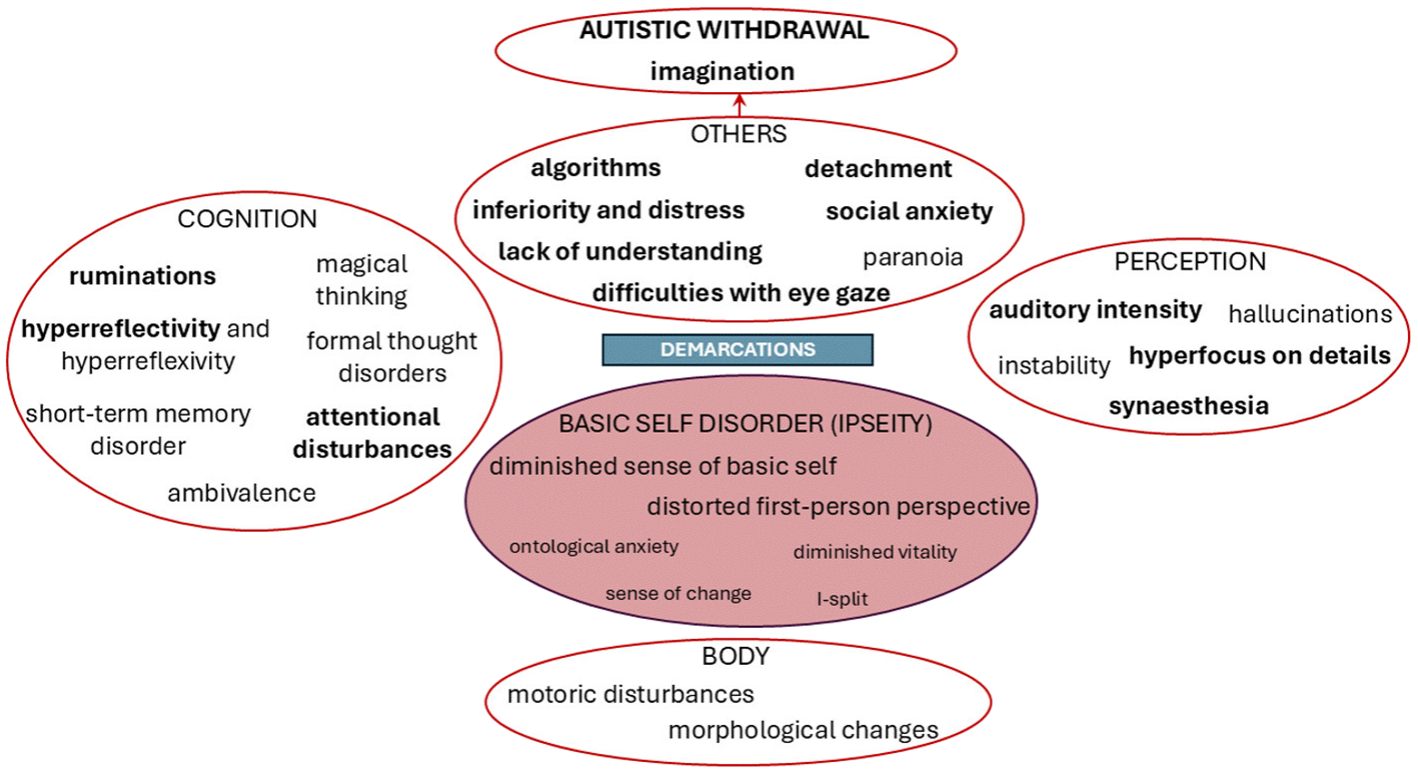
Central characteristics of schizophrenia spectrum disorder.

**Figure 4:**
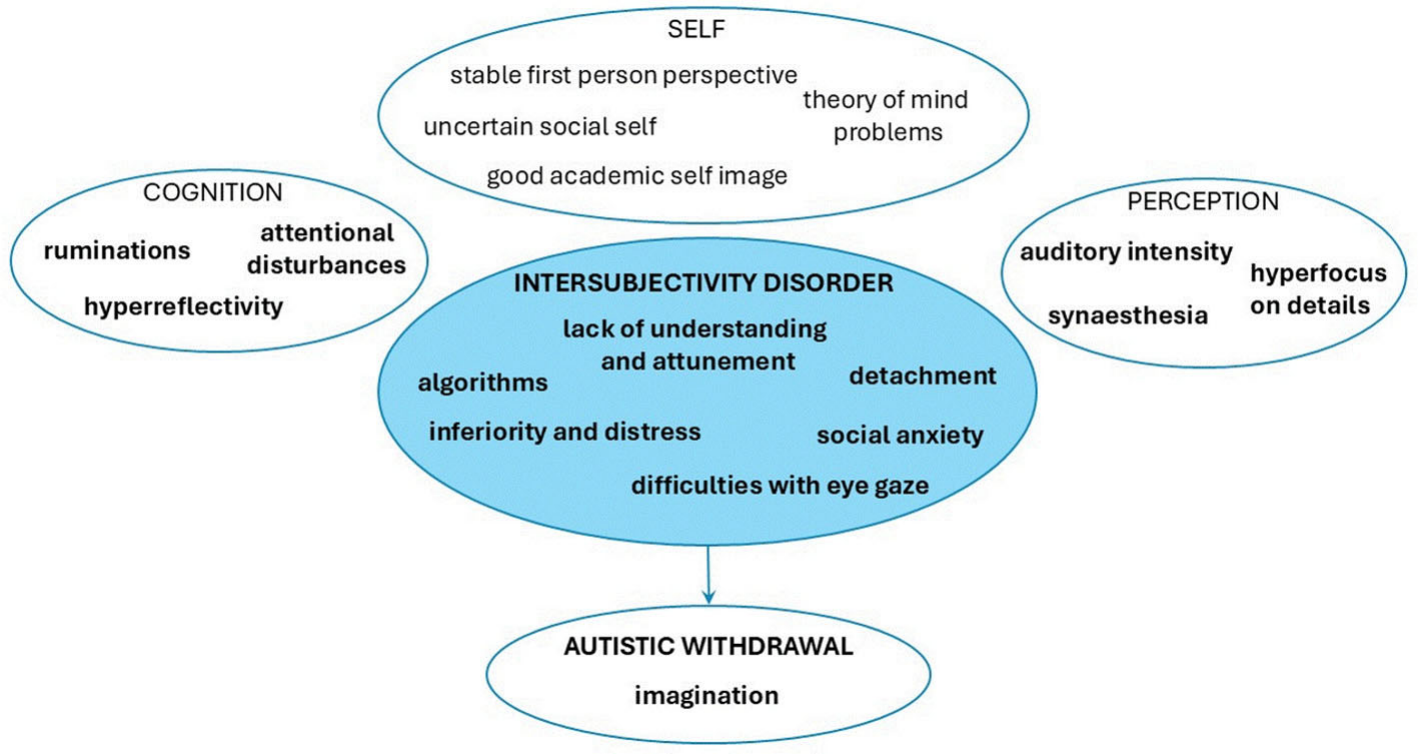
Central characteristics of autism spectrum disorder.

**Figures 3** and **4** show the main characteristics of SSD and ASD obtained in our study. Overlaps are highlighted in bold.

## 4 Discussion

In line with the theory and our hypothesis, our study showed great overlaps between the two spectra of disorders in the social domain, which can be best described as disturbances of intersubjectivity. Despite these overlaps, we were able to recognize some important differences, which seem likely to be linked to the underlying mechanisms widely assumed to be central to each disorder. Both spectra show disorders of intersubjectivity; however, the central and most distinguishing abnormality in SSD seems to involve a disorder of ipseity with equiprimordial disturbance of intersubjectivity; while in ASD it seems that primary intersubjectivity is affected in isolation. Below, we present the discussion of phenomenological overlaps and differences between ASD and SSD identified in our research in more detail.

### 4.1 Qualitative overlaps between SSD and ASD

As noted, the study identified phenomenological overlaps between the two disorders in EAWE domain *Other Persons* (**Figure 2**). Subjects from both spectra report a lack of social understanding and interpersonal attunement and feelings of emotional distance from others, which may best be understood as indicating some disorder of primary intersubjectivity or basic disorders of being-with-others. Descriptions from both groups indicate a lack of the automatic, spontaneous, or pre-reflective embodied synchrony with others that would supply the social basis of primary common sense (36). The disorder of primary intersubjectivity seems, however, to be even more prevalent in the ASD sample, where all individuals report what seem to be primary social deficits. To view this as a central deficit in ASD disorder is consistent with theoretical research (39). Individuals with ASD often describe the social sphere as a “parallel world” into which they can never fully penetrate, stating that no matter how hard they try, a basic disconnect between them and others always exists. This permanent social gap can be understood through a basic deficit of emotional reciprocity with others, as discussed in Kanner’s pioneering article (3). Deficits in embodied interaffectivity (50) are replaced by compensatory intellectual strategies and algorithms to grasp the social rules.

Individuals from both samples are prone to hyper-reflection, where they try to intellectually understand everyday life principles that are usually implicitly known. Hyper-reflection as a compensatory strategy has a rational or philosophical quality of thinking, and individuals from both samples spontaneously describe themselves as “eternal philosophers”. At this point we can differentiate hyper-reflection from more primary or “operative” forms of hyperreflexivity—the latter involving an almost automatic tendency to focus on inner sensations that would normally stay implicit or unnoticed (51). The latter are more prominent in SSD and can be understood as a more direct manifestation of ipseity disorder.

In social relationships, subjects from both samples feel inferior, anxious, and ruminate or replay social scenes that happened to them during the day. To be accepted among others, they sometimes try to hyper-adapt via tending to agree with others in an almost automatic or compulsive fashion. In both samples, overlaps are also reflected in poor eye contact. Our interviews suggested, however, that whereas poor eye contact in ASD is associated with a disorder of intersubjectivity, in which the use of eye contact lacks spontaneity and naturalness, poor eye contact in SSD is mostly associated with feelings of demarcation-disturbance or lack of ego boundaries—what Bleuler termed transitivity (1).

Both of our samples were expressive of “autism” or active social withdrawal due to social discomfort and anxiety. Autism in both samples, according to their reports, is filled with imagination and fantasy. However, for individuals with SSD the reason for active withdrawal lies in demarcation disturbance, while for individuals with ASD it lies in exhaustion following their efforts to interact with others. Individuals with ASD described their withdrawal as “refuge”, while an SSD individual described withdrawal as “an opportunity to create internal films.”

Further important overlaps involved synesthesia and increased intensity of auditory perceptions, known as hypersensitivity in ASD and listed in diagnostic criteria for ASD (52). Deviations in the sensory/perceptual domain in both spectra indicate abnormalities at the neurological level, which can significantly interfere with the development of embodied social perception in both disorders (36). Attention disorders, with excessive focus on details, are recognized in both samples, which can indirectly indicate problems in holistic perception or multisensory integration (53). Deficits in recognizing the whole, i.e. gestalt perception, are recognized in ASD and are described in accord with the notion of Central Coherence Disorder (47). Changes in the perceptual field in schizophrenia may even represent a core impairment (53), with reduction in the perceptual organization that would normally serve as the “pre-reflective basis” for action and cognition.

### 4.2 Qualitative differences between SSD and ASD

Minimal self-disorder or ipseity disturbance appears as an important differential diagnostic phenomenon between SSD and ASD, with the disorder proving to be a prototypical characteristic for SSD, which is consistent with similar empirical research (29). Ipseity disturbance is generally closely connected with corporeality, since the lived body is implicitly present in every feeling, perception and action, thus mediating our everyday being-in-the-world and being-with-others (40). This deep experiential quality of self-uncertainties about individual’s humanity, sex and/or age is only present in SSD sample, for example: “*Sometimes I feel like I am not here, like I am another person inside, or a cat.”*

The main problem in ASD is their interpersonal or social self, which is manifested in constant feelings of being different among others, described by one subject as feeling like *“an alien, who landed on the wrong planet”*. However, their first-person perspective is solid, as verbalized by one individual with ASD: *“I am very present to myself.”* Individuals from the ASD sample have difficulties with the third-person perspective, finding it hard to see themselves through the eyes of others, best described by the deficits in the Theory of Mind (54).

Another important differential feature obtained in the study is the disturbance of demarcation or self-other distinction. Being conscious of another consciousness may threaten the SSD patient with a loss of her or his self (36). This differential characteristic between disorders, already highlighted within theoretical phenomenological research on differences between disorders (39), is further confirmed by our study. The disturbances of demarcation in SSD reported in our study were reflected in experiences of threatening physical touch, discomfort with eye contact, and in social withdrawal; meanwhile individuals with ASD reported uneasiness in social contacts without fear of being invaded.

Paranoid anxiety and ideations significantly differentiate the samples in our study. Individuals with ASD, who in some reports do know paranoid like ideas following negative experiences in social situation (35), in our sample did not report explicit paranoid ideas. They said that their social anxiety is related, not to feelings that others may be threatening, but to feelings of confusion in certain social situations and fear of appearing strange: e.g., *“In a crowd, I feel uncomfortable, but I’m not afraid that I might be robbed or something like that, I don’t feel that I could be attacked.”* Essentially, individuals with ASD seem to have firm, impermeable boundaries between themselves and others, sometimes to the point that others are not even noticed, as verbalized by one individual with ASD: *“I finally noticed in high school that there are others, that they socialize,—I just read my books, and that was my world.”*

Another important differential diagnostic feature is magical thinking, described only by people with SSD in our study. In reports of ASD individuals, we could recognize that they cling to external structures and rules understood as a compensatory mechanism due to their profound lack of social common sense and consequent confusion in social situations. Even in their descriptions of imagination a pattern is recognizable - their fantasy world is always created “on something” within pre-established drafts. A prototypical example of an individual with ASD: *“My imagination is like a simulation; I repeat stories I read, erase characters and place my own.”*

An important difference in the field of perception is that individuals from the SSD sample report instability of perception, with objects seen or voices heard seeming to move or change (which also represents an obstacle for some SSD patients in socialization). Hallucinations were not reported in the ASD sample.

In the field of cognition, short-term memory disorders appear only in the SSD sample, a core neuropsychological impairment in patients with schizophrenia (55).

## 5 Strengths and limitations

In the present paper, we present empirical findings and analysis comparing SSD and ASD. Our focus is on the first-person perspective of patients, which may offer significant differential diagnostic insights with psychotherapeutic potential for both disorders. It also represents clinical and phenomenological exploration and an insight into the world of people with ASD, which still represents an undiscovered area (34). Empirical phenomenological exploration of the ASD world gives an opportunity to transparently explain and understand features of ASD as being in intimate developmental relationship with each other with its base in primary intersubjectivity deficits (56). Our research contributes additional value to theoretical phenomenological research in comparison between SSD and ASD disorders.

We would like to acknowledge that by using EASE and EAWE, some of the finer nuances of subjective experiences in people with ASD could have been overlooked. This is because both interview formats (and especially the EASE) were primarily constructed to investigate subjective experiences in patients with schizophrenia. We also hypothesize that additional differences between the two spectra might show up in the overall EAWE scores for domains that were excluded in this study: namely, Temporality, Language (referring here to the subjective *experience* of language), and Atmosphere. It should be recalled that the two EAWE domains that *were* used (Objects and space, and Persons) were selected because of the expectation of much overall overlapping between SSD and ASD on these dimensions. We did, however, also consider possible underlying qualitative differences between the two spectra within each of these two selected domains.

Another limitation worth highlighting is the sample size (21/21), which is relatively small and less reliable in terms of generalizing the obtained results. However, the study is fundamentally qualitative in nature, where the number of interviews or data points required to reach saturation is not fixed (57). In our research, we worked with two homogeneous samples, where the accuracy of the diagnosis was verified twice. The interviews were conducted using a systematic phenomenological approach, with predefined research themes. The participants’ experiences consistently emerged throughout the study, providing rich, meaningful insights that enabled critical conclusions.

## 6 Conclusion

Our study highlights the importance of research eliciting first-person perspective as a complement to third-person approaches, which may not capture the full picture of a specific disorder. By examining and analyzing the subjective experiences of individuals with SSD and ASD in parallel, we have identified key qualitative differences that are related to the centrality of the specific disorder and illuminate differences despite overlapping behaviors. Both disorders overlap in deeper deficits in social functioning, which are best described as disturbance of ipseity in the case of SSD and disturbance of primary intersubjectivity in the case of ASD. In SSD, we thus encounter pervasive self-uncertainties and diminished self-presence with difficulties with demarcation or self-other distinction and paranoid fear of being invaded. In ASD, the most significant disturbance seems to involve primary intersubjectivity, which gives rise to compensatory strategies and ways of being that tend to be explicit, stereotyped, and repetitive. This is reflected in their systematic thinking, framed fantasy, in an exaggerated first-person presence and reference, and in understanding others through compensatory algorithmic strategies.

Clinical phenomenological research has proven to be an invaluable method for exploring the subjective experiences of individuals with ASD. Contrary to the assumption that people with ASD lack introspective abilities, our research demonstrates that their inner experiences are rich and can be vividly described. This finding challenges the notion of ASD individuals as an “empty fortress” (58), revealing instead a complex inner world “full of fragility” (37). Further detailed phenomenological studies on ASD, following the tradition of systematic phenomenological research on SSD, would be highly beneficial. In future phenomenological studies of ASD, it would be meaningful to place greater emphasis on the specific exploration of individual domains of psychological functioning (e.g., experience of self, bodily experience, perception, others, etc.—as systematically categorized in EASE or EAWE). This would deepen our understanding of the disorder and highlight important qualitative differences between ASD and other disorders that involve social difficulties. Systematic qualitative research on ASD could also aid in differentiating specific subcategories within the spectrum that share underlying commonalities, with the aim of providing more specifically targeted support.

## Data Availability

The original contributions presented in the study are included in the article/supplementary material. Further inquiries can be directed to the corresponding author.
